# Association between acute kidney injury and norepinephrine use following cardiac surgery: a retrospective propensity score-weighted analysis

**DOI:** 10.1186/s13613-022-01037-1

**Published:** 2022-07-04

**Authors:** Pierre Huette, Mouhamed Djahoum Moussa, Christophe Beyls, Pierre-Grégoire Guinot, Mathieu Guilbart, Patricia Besserve, Mehdi Bouhlal, Sarah Mounjid, Hervé Dupont, Yazine Mahjoub, Audrey Michaud, Osama Abou-Arab

**Affiliations:** 1 Department of Anesthesiology and Critical Care Medicine, Amiens Picardy University Hospital, 80054 Amiens, France; 2 Anesthesia and Critical Care Department, Institut Coeur-Poumon, Lille Hospital University, 59000 Lille, France; 3 Department of Anesthesiology and Critical Care Medicine, Dijon University Hospital, 21000 Dijon, France; 4 Department of Biostatistics, Amiens Picardy University Hospital, 80054 Amiens, France

**Keywords:** Acute kidney injury, Mortality, Cardiac surgery, Norepinephrine, Propensity analysis

## Abstract

**Background:**

Excess exposure to norepinephrine can compromise microcirculation and organ function. We aimed to assess the association between norepinephrine exposure and acute kidney injury (AKI) and intensive care unit (ICU) mortality after cardiac surgery.

**Methods:**

This retrospective observational study included adult patients who underwent cardiac surgery under cardiopulmonary bypass from January 1, 2008, to December 31, 2017, at the Amiens University Hospital in France. The primary exposure variable was postoperative norepinephrine during the ICU stay and the primary endpoint was the presence of AKI. The secondary endpoint was in-ICU mortality. As the cohort was nonrandom, inverse probability weighting (IPW) derived from propensity scores was used to reduce imbalances in the pre- and intra-operative characteristics.

**Results:**

Among a population of 5053 patients, 1605 (32%) were exposed to norepinephrine following cardiac surgery. Before weighting, the prevalence of AKI was 25% and ICU mortality 10% for patients exposed to norepinephrine. Exposure to norepinephrine was estimated to be significantly associated with AKI by a factor of 1.95 (95% confidence interval, 1.63–2.34%; *P* < 0.001) in the IPW cohort and with in-ICU mortality by a factor of 1.54 (95% confidence interval, 1.19–1.99%; *P* < 0.001).

**Conclusion:**

Norepinephrine was associated with AKI and in-ICU mortality following cardiac surgery. While these results discourage norepinephrine use for vasoplegic syndrome in cardiac surgery, prospective investigations are needed to substantiate findings and to suggest alternative strategies for organ protection.

**Supplementary Information:**

The online version contains supplementary material available at 10.1186/s13613-022-01037-1.

## Background

Vasoplegic syndrome is frequent following cardiac surgery [1]. The mechanism is multifactorial, but is mostly induced by injuries due to cardiopulmonary bypass and ischemia reperfusion. Vasoplegic syndrome is characterized by a decrease in organ perfusion, which can lead to postoperative organ failure. Hence, the administration of a vasopressor is required to preserve organ perfusion. Norepinephrine is the most common vasopressor used in cardiac surgery. Based on a large study from North America, one-third of patients receive norepinephrine on the first day after surgery [2]. There are few high-evidence studies to actually support which vasopressor to use in the first line. Norepinephrine restores arterial pressure, but can compromise oxygen delivery to the organs [3]. In the sole randomized study on vasopressor use in cardiac surgery, the administration of a vasopressin analog versus norepinephrine reduced postoperative complications, notably the incidence of acute kidney injury (AKI) [4]. Thus, the challenge is to counter organ hypoperfusion with norepinephrine use, which can potentially increase the frequency of cardiac events and compromise microcirculation [5–7]. In a recent expert consensus statement, the authors unanimously recommended the use of norepinephrine in the first line [8]. However, as mentioned in the expert statement, supporting evidence is still scarce.

Here, we aimed to assess the association between norepinephrine exposure and adverse outcomes following cardiac surgery. We specifically focused on AKI and in-intensive care unit (ICU) mortality following cardiac surgery. Previous studies have suffered from major confounding factors, leading to difficulties in interpreting outcomes. Here, we used a propensity score-weighted analysis. Such analysis allows the proper adjustment of confounding factors with the advantage of retaining most of the observations [9].

The primary endpoint was the estimation of the AKI risk according to norepinephrine exposure. The secondary endpoint was the estimation of in-ICU mortality according to norepinephrine exposure.

## Materials and methods

### Ethics

This retrospective observational study included all consecutive patients admitted for cardiac surgery under cardiopulmonary bypass at Amiens Hospital University in France from January 1, 2008, to December 31, 2018. According to French laws on medical research, the study falls within the scope of French Reference Methodology MR-004 (declaration identifier: DRCI_ANESTHREA_ABOUARAB, declaration on the 8th of October 2018) [10]. The study did not require informing or consent of the patients. No identification or nominative data of the patients were collected. Reporting is according to a guideline on reports of propensity score analysis [11].

### Study population

The study population consisted of patients admitted to our center for cardiac surgery under cardiopulmonary bypass.

The non-inclusion criteria were beating-heart surgery, refractory CPB weaning with the requirement for a circulatory device, non-cardiac surgery, nonscheduled cardiac surgery (active endocarditis, aortic dissection), exposure to other vasoactive drugs (levosimendan, dobutamine, epinephrine) and active postoperative bleeding (requirement for more than four red blood cell units or redo surgery for revision of coagulation), patient with a medical history of pulmonary hypertension.

### Data extraction

Data were collected from our institutional database using the French classification for medical procedures “Classification Commune des Actes Médicaux” (CCAM). Each diagnosis (outcome) was associated with a unique diagnostic code number. The coding of medical acts and diagnoses in our database was carried out by the same physician.

We performed computerized data extraction from our local data system DxCare® (DxCare®, Medasys) and Clinisoft® (Centricity Critical Care Clinisoft®, GE Healthcare), the two computerized systems we have been using since 2007.

### Data collection and definition of acute kidney injury

We collected the following data from our local database: age, gender, body-mass index, medical history (hypertension, coronary disease, diabetes, dyslipidemia, chronic kidney disease, peripheral vascular disease), the type of surgery (coronary bypass graft, valve surgery, combined surgery, and other surgery), the duration of CPB, and the duration of the aortic clamp.

The following postoperative data were collected: Simplified Acute Physiology Score II (SAPS II) at ICU admission, cumulative dose of norepinephrine within 48 h after admission (mg), cumulative diuresis at day 1 and day 2 (ml), cumulative dose of crystalloid expansion and colloid expansion after 24 and 48 h (ml), creatinine (µmol l^−1^) at ICU admission/day 1/day 2, arterial partial pressure in oxygen (PaO_2_, expressed in mmHg) at ICU admission/day 1/day 2, aspartate-amino-transferase (ASAT) at ICU admission/day 1/day 2, and the duration of ICU stay (days).

AKI was defined according to the KDIGO criteria guidelines as an increase in creatinine > 26.5 µmol l^−1^ or diuresis < 0.5 ml kg^−1^ h^−1^ within 48 h [12].

In-ICU mortality was defined as the occurrence of death during the first stay in the ICU immediately after the surgery.

### Endpoints

The primary endpoint was the occurrence of AKI during the ICU stay after cardiac surgery. The secondary endpoint was the rate of in-ICU mortality.

### Statistical analysis

Patient characteristics are described overall and by group (exposed to norepinephrine [N+] and not exposed to norepinephrine [N-]). Quantitative data are reported as means and standard deviations, if normally distributed, and as medians and interquartile ranges (IQR) if non-normally distributed. Data were compared using Mann–Whitney–Wilcoxon or Student *t* tests, as appropriate. Qualitative data are described as absolute numbers and percentages, and groups were compared using Pearson’s χ^2^ or Fisher’s exact tests, as appropriate. Missing data are reported and were imputed by predictive mean imputation (pmm). The following prognostic variables related to the outcome at *P* < 0.2 in univariate analysis were included in the propensity score (regardless of the differences between the two groups) (see Additional file 1: Table S1 and Additional file 2: Table S2): age, gender, BMI, history of hypertension, coronary disease, diabetes, dyslipidemia, chronic kidney disease or peripheral vascular disease, creatinine, cardiopulmonary bypass, duration of the aortic clamp, type of surgery, and SAPS-II [13, 14]. Baseline platelets, baseline hemoglobin and intake of inotropes (adrenaline or dobutamine) at 48 h were forced in the propensity score. The probability of being exposed to norepinephrine (regardless of the differences between the two groups) was estimated using a logistic regression for each patient. For ATE (average treatment effect on the entire population) analysis, weights were attributed to each patient of the N+ and N− groups, making the two groups similar for the variables in the propensity score. These weights were calculated using stabilized inverse probability of treatment weighting (SIPTW). The positivity hypothesis of the propensity score (existence of a common support between the two groups) and balance (standardized mean differences < 10%) were assessed for each outcome [15]. Outcomes (renal failure and death) are reported as percentages and 95% confidence intervals after weighting. Odds ratios (ORs) were estimated after weighting on the propensity score and compared using Pearson’s χ^2^ test. All tests were two-sided, with a significance level of 5%. Statistical analyses were performed using R software, version 4.0.4. ©2021, with the « tableone», « survey», « mice», « ggplot2», « car», « questionr», « weights», « WeightIt», « cobalt», and « epiR» packages.

## Results

### *Study population *(Fig. 1)

**Fig. 1:**
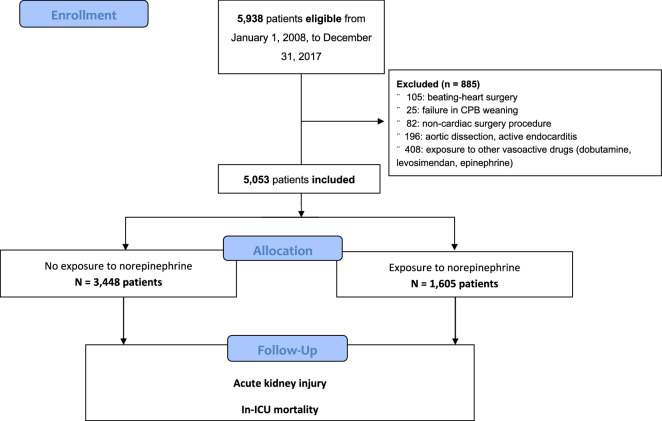
Flowchart of participants. CPB: cardiopulmonary bypass, ICU: intensive care unit

In total, 5938 patients from January 1, 2008, to December 31, 2017, were initially selected from the database. Among them, 885 were excluded: 105 because of beating-heart surgery, 25 because of being refractory to CPB weaning with the requirement of a circulatory support device, 82 for a non-cardiac procedure under CPB, 196 because of nonscheduled surgery (aortic dissection or active endocarditis), and 408 because of exposure to other drugs or because of active bleeding. Finally, the data of 5053 patients were analyzed.

### Baseline characteristics (Table 1)

**Table 1:** Patient characteristics of the cohort

Variables	Total cohort (*n* = 5053)	No exposure to norepinephrine (*n* = 3448)	Exposure to norepinephrine (*n* = 1605)	*P* value	SMD
*Preoperative*					
Age, years	69 [60- 76]	68 [60- 76]	70 [61–77]	**< 0.001**	0.121
Male gender, *n *(%)	3487 (69)	2407 (70)	1080 (67)	0.069	0.055
BMI, kg m^−2^	27.4 [24.3–30.7]	27.6 [24.5–30.8]	26.8 [23.8–30.5]	**< 0.001**	0.057
Medical history, *n *(%)					
Hypertension	2,815 (56)	1,925 (56)	890 (56)	0.801	0.08
Coronary disease	598 (11)	385 (11)	213 (13)	**0.031**	0.033
Diabetes	984 (20)	690 (20)	294 (18)	0.157	0.043
Dyslipidemia	381 (8)	274 (8)	107 (7)	0.109	0.049
Chronic kidney disease	224 (4)	126 (4)	98 (6)	**< 0.001**	0.114
Peripheral vascular disease	264 (5)	188 (6)	76 (5)	0.286	0.033
Creatinine, µmol l^−*1*^	77 ± 9	79 ± 10	77 ± 10	0.932	0.472
Hemoglobin*, *g dl^−1^	12.3 ± 0.4	12.3 ± 0.3	12.6 ± 0.4	0.629	0.682
*Intraoperative*					
CPB time, *n *(%)	94 [65–128]	89 [63–122]	105 [74–140]	**< 0.001**	0.331
Aortic clamp time, *n *(%)	64 [43–90]	61 [41–87]	69 [46–96]	**< 0.001**	0.098
Surgery type, *n *(%)					
CABG	1,430 (28)	962 (28)	468 (29)	**< 0.001**	0.147
Valve surgery	1,874 (37)	1,254 (36)	620 (39)		
Combined surgery	548 (11)	336 (10)	212 (13)		
Other	1,201 (24)	896 (26)	305 (19)		
*Postoperative*					
SAPS II at ICU admission	35 [30–43]	34 [28–40]	40 [33–48]	< 0.001	0.603
Cumulative dose of norepinephrine during 48 h, *mg*	–	–	6.9 ± 0.4	–	–
Cumulative diuresis, ml					
Day 1	1370 [1030–1821]	1409 [1086–1845]	1280 [850–1748]	< 0.001	0.165
Day 2	2650 [2110–3320]	2690 [2165–3345]	2570 [1965–3275]	< 0.001	0.105
Cumulative colloid expansion after 48 h; ml	250 [0–500]	250 [0–500]	500 [0–750]	< 0.001	0.304
Cumulative crystalloid expansion after 48 h, ml	1306 [990–1647]	1274 [956–1575]	1422 [1058–1807]	< 0.001	0.412
Creatinine; µmol l^−1^					
Post CPB	79 [65–97]	77 [64–94]	82 [67–105]	< 0.001	0.189
Day 1	88 [69–121]	84 [67–111]	100 [74–143]	< 0.001	0.310
Day 2	80 [63–116]	76 [61–104]	92 [68–154]	< 0.001	0.356
PaO_2_, mmHg					
Post CPB	183 [52–292]	189 [54–296]	171 [49–282]	< 0.001	0.092
Day 1	108 [73–144]	108 [73- 143]	109 [73–146]	0.32	0.031
Day 2	88 [71–113]	88 [71–113]	89 [70–115]	0.996	0.356
ASAT, UI					
Post CPB	50 [35–73]	48 [34- 69]	57 [37–84]	< 0.001	0.118
Day 1	64 [43–102]	60 [42–92]	74 [47–130]	< 0.001	0.115
Day 2	54 [36–90]	51 [35–80]	64 [39–119]	< 0.001	0.150
ICU stay, days	3 [2–4]	3 [2–4]	3 [2–6]	< 0.001	0.301

Data are presented as medians [interquartile ranges] or numbers (percentages). BMI: body-mass index, CPB: cardiopulmonary bypass, CABG: cardiopulmonary bypass, ICU: intensive care unit, CPB: cardiopulmonary bypass, SAPS II: Simplified Acute Physiology Score II, ASAT: aspartate-amino-transferase, SMD: mean standardized difference

The median age of the study population was 69 [60–76] years and it was predominantly male (69%). The main interventions were coronary artery bypass graft, valve surgery, and combined surgery.

In total, 1605 patients were exposed to norepinephrine, representing a rate of 32%. Patients receiving norepinephrine were significantly older, with a medical history of more coronary disease and more chronic kidney disease. In terms of intraoperative characteristics, patients exposed to norepinephrine had a significantly longer duration of CPB and aortic clamp. The rate of combined surgery was also higher for this group of patients.

Among patients with others vasoactive drugs, 392 were exposed to dobutamine, 12 to epinephrine and 3 to levosimendan. The patients’ baseline and intraoperative characteristics are reported in a supplementary table (Additional file 3: Table S3).

### Comparison of hemodynamics and biological parameters during the ICU stay (Table 1)

During the ICU stay, the cumulative dose of fluid expansion was significantly greater for patients exposed to norepinephrine after 48 h of postoperative care for both crystalloid and colloid solutions. In terms of kidney and hepatic monitoring, creatinine and ASAT levels were significantly higher for patients exposed to norepinephrine at ICU admission and day 1 and day 2 after cardiac surgery. PaO_2_ was significantly lower for patients exposed to norepinephrine at ICU admission but was similar on days 1 and 2. The median duration of the ICU stay was longer for patients exposed to norepinephrine.

**Fig. 2:**
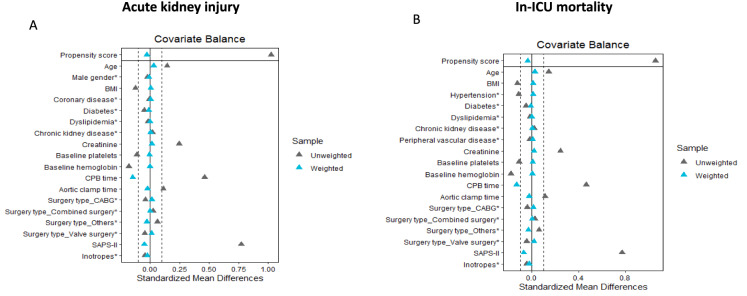
Love plots for standardized mean differences comparing covariate values before (grey triangle) and after (blue triangle) propensity score weighting for the assessment of acute kidney injury (**A**) and in-ICU mortality (**B**). Standardized mean differences are expressed as percentages. An absolute MSD < 15% was considered to support the assumption of balance between the groups. BMI: body-mass index, CABG: coronary bypass graft, CPB: cardiopulmonary bypass, SAPS II: Simplified Acute Physiology Score. *: inotropes include the use of dobutamine or epinephrine associated or not to norepinephrine

After IPW for the assessment of AKI and in-hospital mortality, the mean standardized difference was < 15% for pre- and intra-operative variables (Tables 2 and 3). We also report the variables in a Love plot for AKI (Fig. 2A) and in-ICU mortality (Fig. 2B).

**Table 2:** Imbalance of patient characteristics before and after propensity weighting in the assessment of acute kidney injury

Variables	Before weighting			After weighting		
	No exposure to NE (*n* = 3448)	Exposure to NE (*n* = 1605)	SMD	No exposure to NE (*n* = 3498)	Exposure to NE (*n* = 1581)	SMD
Age (years)	68 [60–76]	70 [61,7]	0.120	69 [61, 76]	68 [60, 76]	< 0.001
Male gender	2407 (70)	1080 (67)	0.054	2429 (69)	1105 (70)	0.009
BMI (kg m^−2^)	27.48 [24.44–30.72]	26.83 [23.83–30.42]	0.017	27.53 [24.39, 30.76]	26.76 [23.72–30.26]	0.025
Hypertension	1925 (56)	890 (56)	0.008	1922 (55)	868 (55)	0.001
Coronary disease	385 (11)	213 (13)	0.064	404 (12)	189 (12)	0.012
Diabetes	690 (20)	294 (18)	0.043	672 (19)	304 (19)	< 0.001
Dyslipidemia	274 (8)	107 (7)	0.049	259.5 (7)	122 (8)	0.010
Chronic kidney disease	126 (4)	98 (6)	0.114	155.9 (5)	75 (5)	0.013
Peripheral vascular disease	188 (6)	76 (5)	0.033	206 (6)	74 (5)	0.055
Hemoglobin, g dl^−1^	12.5 ± 0.5	12.2 ± 0.1	0.181	12.3 ± 0.7	12.3 ± 0.8	0.002
Platelet, 10^3^ mm^−3^	148 ± 34	139 ± 35	0.111	146 ± 34	143 ± 36	0.004
Creatinine (µ mol l^−1^)	77 [64–94]	82 [67–105]	0.187	79 [65–98.09]	78 [65–97.34]	0.013
CPB time (min)	84 [56–118]	100 [67, 136]	0.336	88 [58–125]	91 [61–125]	0.032
Aortic clamp time (min)	57 [35–83]	64 [42–94]	0.111	59 [37–87]	60 [39–89]	0.004
Surgery type						
CABG	962 (28)	468 (29)	0.185	1000 (29)	461 (29)	0.012
Valve surgery	1254 (36)	620 (39)		1285 (37)	576 (36)	
Combined surgery	336 (10)	212 (13)		369 (11)	166 (11)	
Others	896 (26)	305 (19)		845 (24)	379 (24)	
SAPS II	34 [28–40]	40 [33–48]	0.595	35 [29–43]	36 [30–43]	0.026
Inotropes	0 (0)	128 (8)	0.306	0 (0)	87 (6)	0.226

An absolute MSD < 10% was considered to support the assumption of a balance between the groups. SMD: standardized mean differences. Data are presented as medians [interquartile ranges] or as numbers (percentages). NE: norepinephrine, CABG: coronary bypass graft, CPB: coronary bypass, SAPS II: Simplified Acute Physiology Score. BMI, hypertension, and peripheral vascular disease were not selected for the propensity weighting as the P value for their association with acute kidney injury was over 20%

**Table 3:** Imbalance of patient characteristics before and after propensity weighting in the assessment of in-ICU mortality

Variables	Before weighting			After weighting		
	No exposure to NE (*n* = 3448)	Exposure to NE (*n* = 1605)	SMD	No exposure to NE (*n* = 3502)	Exposure to NE (*n* = 1582)	SMD
Age, years	68 [60–76]	70 [61–77]	0.120	69 [61–76]	69 [61–76]	0.002
Male gender	2407 (67)	1080 (67)	0.054	2456 (70)	2456 (70)	0.036
BMI, kg m^−2^	27.48 [24.44–30.72]	26.83 [23.83–30.42]	0.017	27.50 [24.39–30.73]	27.50 [24.39–30.73]	0.005
Hypertension	1925 (56)	890 (56)	0.008	1912 (55)	1919 (55)	0.018
Coronary disease	385 (11.2)	213 (13)	0.064	380 (11)	381 (11)	0.078
Diabetes	690 (20)	294 (18)	0.043	671 (19)	671 (19)	0.003
Dyslipidemia	274 (8)	107 (7)	0.049	259 (7)	258.7 (7)	0.007
Chronic kidney disease	126 (4)	98 (6)	0.114	157 (5)	157 (5)	0.022
Peripheral vascular disease	188 (6)	76 (5)	0.033	187 (5)	187 (5)	0.018
Hemoglobin, g dl^−1^	11.52 ± 1.54	11.23 ± 1.64	0.181	11.3 ± 1.56	11.4 ± 1.43	0.004
Platelet, 10^3^ mm^−3^	157 ± 58	150 ± 66	0.111	146 ± 34	144 ± 35	0.007
Creatinine, µ mol l^−1^	77 [64–94]	82 [67–105]	0.187	79 [65–99]	79 [65–99]	0.012
CPB time, min	84 [56–118]	100 [67–136]	0.336	88 [58–125]	88 [58–125]	0.036
Aortic clamp time, min	57 [35–83]	64 [42–94]	0.111	59 [37–87]	59 [37–87]	0.005
Surgery type			0.185			
CABG	962 (28)	468 (29)		1002 (29)	1002 (29)	0.010
Valve surgery	1254 (36)	620 (39)		1284 (37)	1284 (37)	
Combined surgery	336 (10)	212 (13)		369 (11)	369 (11)	
Others	896 (26)	305 (19)		847 (24)	847 (24)	
SAPS II	34 [28–40]	40 [33–48]	0.595	35 [30–43]	35 [30–43]	0.030
Inotropes	0 (0)	128 (8)	0.306	0 (0)	74 (5)	0.223

An absolute MSD < 10% was considered to support the assumption of a balance between the groups. SMD: standardized mean differences. Data are presented as medians [interquartile ranges] or as numbers (percentages). NE: norepinephrine, CABG: coronary bypass graft, CPB: coronary bypass, SAPS II: Simplified Acute Physiology Score. Male gender and coronary disease were not selected for the propensity weighting as the *P* value for their association with acute kidney injury was over 20%

### Association between exposure to norepinephrine and the endpoints (Table 4)

Before weighting, the prevalence of AKI was significantly higher for patients exposed to norepinephrine than those who were not (21.0% [95% CI 19.3 to 22.7] vs 5.3% [4.5 to 6.1], *P* < 0.001). The risk of AKI was significantly associated with exposure to norepinephrine, with a relative risk of 4.74 [95% CI 3.91, 5.75], *P* < 0.001. After IPW, the risk of AKI remained significantly associated with exposure to norepinephrine, with a relative risk of 1.95 [95% CI 1.63, 2.34], *P* < 0.001.

Before weighting, the prevalence of in-ICU mortality was significantly higher in patients exposed to norepinephrine than those who were not (10.5% [95% CI 9.2, 11.8] vs 1.3% [2.1, 3.1], *P* < 0.001). The risk of in-ICU mortality was significantly associated with exposure to norepinephrine, with a relative risk of 8.98 [95% CI 6.34, 12.73], *P* < 0.001. After IPW, the risk of AKI remained significantly associated with exposure to norepinephrine, with a relative risk of 1.54 [95% CI 1.19, 1.99], *P* < 0.001.

## Discussion

In this population-based observational propensity score-matched study among cardiac surgery patients, we found that exposure to norepinephrine is associated with a higher risk of AKI and in-ICU mortality. Our results are in accordance with those of previous reports (Table 4).

**Table 4:** Incidence risk for acute kidney injury (AKI) and in-ICU mortality according to exposure to norepinephrine (NE)

Variables	Incidence risk (%)	OR (95% CI)	*P*-value
*Before propensity score-weighted estimation*			
AKI,			
NE exposure	21.0 [19.3 to 22.7]	4.74 (3.91 to 5.75)	< 0.001
In-ICU mortality, NE exposure	10.5 [9.2 to 11.8]	8.98 (6.34 to 12.73)	< 0.001
*After propensity score-weighted estimation*			
AKI,			
NE exposure	14.0 [13.5 to 15.5]	1.95 (1.63 to 2.34)	< 0.001
In-ICU mortality,			
NE exposure	6.0 [5.0 to 7.0]	1.54 (1.19 to 1.99)	< 0.001

CI: confidence interval, OR: odds ratio, ICU: intensive care unit

Ours is the largest observational study, to date, on the association between norepinephrine exposure and postoperative morbi-mortality and the first to use a matched propensity analysis.

Our study had several strengths. Preoperative, intraoperative, and postoperative data were extracted from our local database, reducing the risk of error. The monitoring of diuresis was reported hourly in our electronic record, which is our routine standard care, allowing the precise determination of AKI. In addition, the large amount of collected data, including biological investigations, made it possible to control for numerous cofounders.

Despite their coming from a propensity matched analysis, our results must be interpreted with precaution. Propensity matching was based on available data, but there may still be residual confounding factors. Indeed, we were unable to include intraoperative hemodynamic parameters and the CPB settings. Data on the chronic medication of patients were also missing, notably that on the chronic use of ß-blockers and angiotensin-converting enzyme inhibitors [16, 17].

In addition, the indication of norepinephrine may concern various hemodynamic entities, including refractory vasoplegic syndrome, but also transient hypovolemia and/or cardiogenic shock. Unfortunately, no data were available on the postoperative left ventricular ejection fraction or cardiac output to discriminate between these hemodynamic entities. Nevertheless, we excluded patients who received any inotropic drug (dobutamine, levosimendan, milrinone) from our cohort, assuming that the sole exposure to norepinephrine reflects patients with persistent arterial hypotension. Finally, we found a rate of exposure to norepinephrine of 30%, which is similar to that of the most recent prospective studies [4, 18].

Our findings are not novel and it is well known that a patient with circulatory failure has a higher risk of AKI and mortality. However, previous retrospective reports used less robust statistical methods, such as logistic regression or matching, to adjust for confounding factors relative to a propensity matched analysis. Furthermore, we cannot anticipate a future randomized study to compare norepinephrine versus placebo for obvious ethical considerations.

Norepinephrine is the first-line drug recommended for the management of vasoplegic shock according to the most recent expert guidelines from 2021 and the purpose of our study was not to challenge this recommendation [8]. Our intention was to confirm the association between norepinephrine and outcomes. We do not mean to suggest that norepinephrine is responsible for the increase in AKI and in-ICU mortality. As mentioned in a recent expert guideline on vasopressors use in cardiac surgery, there are available alternatives to norepinephrine, such as vasopressin analogs and there is a rationale for the administration of vasopressin analogs in cardiac surgery. Indeed, a deficit in vasopressin has been established following cardiac surgery and exposure to CPB [19]. Thus, it is necessary to assess which vasopressor can restore organ perfusion without the risk of increasing the morbidity and the mortality by the pharmacological adverse effects of the drug, i.e., the α_1_-agonistic effect of norepinephrine [20]. Thus far, the sole randomized study comparing norepinephrine to a vasopressin analog (VANCS study) showed a benefit of the vasopressin analog over norepinephrine. Current guidelines recommend the use of a vasopressor in the second line when a vasoplegic syndrome is refractory to norepinephrine to enhance the vascular response to catecholamine [8, 21]. The probable next step is to assess a vasopressin analog versus norepinephrine as a first-line treatment for vasoplegic syndrome.

Our study had several classic limitations inherent to retrospective cohorts. As already mentioned, certain data would have been valuable for decreasing the residual of the propensity analysis. In addition, our study only concerned one cardiac surgery, with a bias related to patient management and the physicians’ routine. The long period of 10 years (from 2008 to the end of 2017) implies a potentially too great evolution in practices and the indication of norepinephrine.

However, our descriptive data and outcomes are consistent with those of previous reports. [18] Notably, in our report, patients exposed to norepinephrine presented with the expected characteristics (Table 1). These patients tended to have the stigma of ischemic perfusion injuries (lower PaO_2,_ higher plasma enzyme levels, higher creatinine levels).

## Conclusion

Our findings confirm an association between norepinephrine exposure and adverse outcomes using a matched propensity analysis. Prospective studies are needed to substantiate our findings and to validate potential alternative strategies for the management of vasoplegia after cardiac surgery.

## Supplementary Information

**Additional file 1: Table S1.** Description of baseline characteristics according to AKI (acute kidney injury).

**Additional file 2: Table S2. **Description of baseline characteristics according to in-ICU (intensive care medicine) mortality.

**Additional file 3: Table S3. **Baseline and intraoperative characteristics between patients exposed to others vasoactive drugs (dobutamine, levosimendan and epinephrine) and exposed to norepinephrine.

## Data Availability

Datasets are available from the corresponding author on reasonable request.

## Abbreviations

AKI: Acute kidney injury
CPB: Cardiopulmonary bypass
ICU: Intensive care medicine
IPW: Inverse probability weighting
SAPS II: Simplified Acute Physiology Score II
