# Supplementary material for: Association between acute kidney injury and norepinephrine use following cardiac surgery: a retrospective propensity score-weighted analysis
Source: Ann Intensive Care. 2022 Jul 4;12:61. doi: 10.1186/s13613-022-01037-1 (PMC9250911; doi:10.1186/s13613-022-01037-1)
Supplement: Supplementary file 1 — Additional file 1: Table S1. Description of baseline characteristics according to AKI (acute kidney injury). [file 13613_2022_1037_MOESM1_ESM.docx]

**Table S1. Description of baseline characteristics according to AKI (acute kidney injury).** Data are presented as medians [interquartile ranges] or numbers (proportions). **BMI:** body-mass index, **CABG:** coronary bypass graft, **CPB:** cardiopulmonary bypass, **SAPS II:** Simplified Acute Physiology Score II. Inotrope includes dobutamine or epinephrine use ± norepinephrine. Variables with a P value over 20% were not selected for the propensity weighting.

| **Variables** | **No AKI**  **(n = 4,452)** | **AKI**  **(n = 601)** | ***P*-value** |
| --- | --- | --- | --- |
| Age, *years* | 68 [60-76] | 71 [64-78] | < 0.001 |
| Male gender | 3,093 (70) | 394 (66) | 0.057 |
| BMI, *kg m^-2^* | 27.4 [24.2-30.5] | 27.2 [23.9-31.4] | 0.618 |
| Hypertension | 2478 (58) | 337 (56) | 0.883 |
| Coronary disease | 541 (12) | 57 (10) | 0.067 |
| Diabetes | 887 (20) | 97 (16) | 0.032 |
| Dyslipidemia | 351 (8) | 30 (5.0) | 0.015 |
| Chronic kidney disease | 153 (3) | 71 (11.8) | <0.001 |
| Peripheral vascular disease | 232 (5) | 32 (5.3) | 0.984 |
| Hemoglobin, *g dl^-1^* | 12.4 [11.8-12.7] | 11.3 [10.2-12.4] | <0.001 |
| Platelet count, *10^3^/mm^3^* | 148 [120-182] | 139 [112-174] | <0.001 |
| Creatinine | 76 [64-92] | 109 [85-148] | <0.001 |
| CPB time, *min* | 86 [56-119] | 120 [79-164] | < 0.001 |
| Aortic clamp time, *min* | 58 [36-85] | 73 [46-103] | < 0.001 |
| Surgery type |  |  | < 0.001 |
| CABG | 1310 (29) | 120 (20) |  |
| Valve surgery | 1654 (37) | 220 (37) |  |
| Combined surgery | 458 (10) | 90 (15) |  |
| Others | 1030 (23) | 171 (29) |  |
| SAPS-II | 34 [29-41] | 48 [40-57] | < 0.001 |
| Inotrope | 266 (6) | 139 (23) | <0.001 |
