# Supplementary material for: Association between acute kidney injury and norepinephrine use following cardiac surgery: a retrospective propensity score-weighted analysis
Source: Ann Intensive Care. 2022 Jul 4;12:61. doi: 10.1186/s13613-022-01037-1 (PMC9250911; doi:10.1186/s13613-022-01037-1)
Supplement: Supplementary file 2 — Additional file 2: Table S2. Description of baseline characteristics according to in-ICU (intensive care medicine) mortality. [file 13613_2022_1037_MOESM2_ESM.docx]

**Table S2. Description of baseline characteristics according to in-ICU (intensive care medicine) mortality.** Data are presented as medians [interquartile ranges] or numbers (proportions). **BMI:** body-mass index, **CABG:** coronary bypass graft, **CPB:** cardiopulmonary bypass, **SAPS II:** Simplified Acute Physiology Score II. Inotrope includes dobutamine or epinephrine use ± norepinephrine. Variables with a P value over 20% were not selected for the propensity weighting.

| **Variables** | **No in-CU mortality**  **(n = 4,792)** | **In-ICU mortality**  **(n = 261)** | ***P*-value** |
| --- | --- | --- | --- |
| Age, *years* | 69 [60-76] | 72 [63-78] | 0.003 |
| Male gender | 3302 (70) | 185 (71) | 0.546 |
| BMI, *kg min^-1^* | 27.3 [24.2-30.5] | 27.8 [24.2-31.5] | 0.119 |
| Hypertension | 2718 (56) | 97 (37) | < 0.001 |
| Coronary disease | 574 (12) | 24 (9) | 0.209 |
| Diabetes | 947 (20) | 37 (14) | 0.032 |
| Dyslipidemia | 374 (78) | 7 (3) | 0.003 |
| Chronic kidney disease | 196 (4) | 28 (11) | < 0.001 |
| Peripheral vascular disease | 245 (5) | 19 (7) | 0.165 |
| Hemoglobin, *g dl^-1^* | 12.2 [11.4-12.1] | 11.4 [10.8-12.4] | <0.001 |
| Platelet count, *10^3^/mm^3^* | 157 ± 58 | 150 ± 66 | <0.001 |
| Creatinine, *µmol l*^-1^ | 78 [65-95] | 112 [82-157] | < 0.001 |
| CPB time, *min* | 87 [57-120] | 141 [90-196] | < 0.001 |
| Aortic clamp time, *min* | 58 [37-86] | 76 [46-118] | < 0.001 |
| Surgery type, n (%) |  |  | < 0.001 |
| CABG | 1372 (29) | 58 (22) |  |
| Valve surgery | 1798 (38) | 76 (29) |  |
| Combined surgery | 511 (11) | 37 (14) |  |
| Others | 1111 (23) | 90 (35) |  |
| SAPS-II | 35 [29-42] | 54 [44-62] | < 0.001 |
| Inotrope | 293 (6) | 112 (43) | <0.001 |
