# Supplementary material for: Association between acute kidney injury and norepinephrine use following cardiac surgery: a retrospective propensity score-weighted analysis
Source: Ann Intensive Care. 2022 Jul 4;12:61. doi: 10.1186/s13613-022-01037-1 (PMC9250911; doi:10.1186/s13613-022-01037-1)
Supplement: Supplementary file 3 — Additional file 3: Table S3. Baseline and intraoperative characteristics between patients exposed to others vasoactive drugs (dobutamine, levosimendan and epinephrine) and exposed to norepinephrine. [file 13613_2022_1037_MOESM3_ESM.docx]

**Table S3. Baseline and intraoperative characteristics between patients exposed to others vasoactive drugs (dobutamine, levosimendan and epinephrine) and exposed to norepinephrine.** Data are presented as medians [interquartile ranges] or numbers (percentages). **BMI:** body-mass index, **CPB:** cardiopulmonary bypass, **CABG:** cardiopulmonary bypass, ICU: intensive care unit, CPB: cardiopulmonary bypass, **SAPS II**: Simplified Acute Physiology Score II, **ASAT**: aspartate-amino-transferase, SMD: mean standardized difference.

| **Variables** | **Exposure to others vasoactive drugs**  **(n = 408)** | **Exposure to**  **norepinephrine**  **(n = 1,605)** | **P value** |
| --- | --- | --- | --- |
| ***Preoperative*** |  |  |  |
| Age, *years* | 66 [56-75] | 70 [61-77] | **0.032** |
| Male gender, *n (%)* | 273 (69) | 1,080 (67) | 0.742 |
| BMI, *kg m^-2^* | 27.11 [24.22-30.19] | 26.8 [23.8-30.5] | 0.643 |
| Medical history, *n (%)* |  |  |  |
| hypertension | 220 (54) | 890 (56) | 0.842 |
| Coronary disease | 49 (12) | 213 (13) | 0.074 |
| Diabetes | 86 (21) | 294 (18) | 0.359 |
| Dyslipidemia | 20 (5) | 107 (7) | 0.143 |
| Chronic kidney disease | 25 (6) | 98 (6) | 0.428 |
| Peripheral vascular disease | 33 (8) | 76 (5) | **0.042** |
| Creatinine, *µmol l^-1^* | 78 ± 10 | 77 ± 10 | 0.973 |
| Hemoglobin, *g dl^-1^* | 12.5 ± 0.3 | 12.6 ± 0.4 | 0.743 |
| ***Intraoperative*** |  |  |  |
| CPB time, *n (%)* | 127 [90- 153] | 105 [74- 140] | **< 0.001** |
| aortic clamp time, *n (%)* | 83 [51-101] | 69 [46- 96] | **< 0.001** |
| Surgery type, *n (%)* |  |  |  |
| CABG | 73 (18) | 468 (29) | **< 0.001** |
| Valve surgery | 167 (41) | 620 (39) |  |
| Combined surgery | 6 (12) | 212 (13) |  |
| Other | 260 (29) | 305 (19) |  |
|  |  |  |  |
| ***Postoperative*** |  |  |  |
| SAPS II at ICU admission | 41 [34-53] | 40 [33-48] | 0.629 |
| Cumulative dose of norepinephrine during 48 h, *mg* | - | 6.9 ± 0.4 | - |
| Cumulative diuresis, *ml* |  |  |  |
| Day 1 | 1,495 [1,195-1,840] | 1,280 [850-1,748] | 0.323 |
| Day 2 | 2,865 [2,265-3,720] | 2,570 [1,965- 3,275] | 0.218 |
| Cumulative colloid expansion after 48 hours; *ml* | 250 [0- 500] | 500 [0- 750] | **0.023** |
| Cumulative crystalloid expansion after 48 hours, *ml* | 1,237 [843-1,494] | 1,422 [1,058-1,807] | **0.012** |
| Creatinine; *µmol l^-1^* |  |  |  |
| Post CPB | 93 [73-115] | 82 [67-105] | 0.713 |
| Day 1 | 103 [79-145] | 100 [74- 143] | 0.728 |
| Day 2 | 96 [75-138] | 92 [68-154] | 0.927 |
| PaO_2_, *mmHg* |  |  |  |
| Post CPB | 164 [48-290] | 171 [49-282] | 0.372 |
| Day 1 | 110 [66-137] | 109 [73-146] | 0.729 |
| Day 2 | 94 [74-121] | 89 [70-115] | 0.842 |
| ASAT, *UI* |  |  |  |
| Post CPB | 63 [41-98] | 57 [37-84] | 0.429 |
| Day 1 | 83 [58-118] | 74 [47-130] | 0.328 |
| Day 2 | 70 [50-113] | 64 [39-119] | 0.238 |
| ICU stay, *days* | 3 [2-5] | 3 [2-6] | 0.499 |
